# Control of two sucking insect pests, a whitefly ( *Bemisia tabaci* ) and a thrips ( *Frankliniella occidentalis* ), infesting hot peppers by spraying LDH‐formulated dsRNA

**DOI:** 10.1002/ps.70125

**Published:** 2025-08-11

**Authors:** Falguni Khan, Tae‐Geun Song, Yonggyun Kim

**Affiliations:** ^1^ School of Life Sciences, College of Life Sciences and Engineering Gyeongkuk National University Andong Korea

**Keywords:** whitefly, thrips, dsRNA, VIGS, layered double hydroxide, hot pepper

## Abstract

**BACKGROUND:**

Whiteflies and thrips are serious pests by feeding on plant tissues and transmitting plant viruses to crops. To avoid excessive uses of chemical insecticides, spraying double‐stranded RNA (dsRNA) has been proposed as an alternative control measure against these sucking insect pests. However, it is not clear how best to effectively deliver dsRNA to the sucking insects.

**RESULTS:**

A potent dsRNA was generated against a vacuolar type ATPase subunit B (*vATPase‐B*) gene of the silverleaf whitefly, *Bemisia tabaci*, which shares a high sequence homology (85.7%) with that of the western flower thrips, *Frankliniella occidentalis*. The gene was well expressed in the intestine of the whitefly at the nymph and adult stages, in which it was highly expressed at the filter chamber and midgut areas from a fluorescence *in situ* hybridization analysis. Due to its sucking feeding behavior, it was considered impossible to deliver the sprayed dsRNA to the internal body of the sucking insects. This study showed the difference between the two insects, in which the thrips could uptake the sprayed dsRNA and underwent high mortality while the whiteflies could not. To explain the low mortality due to the inefficient dsRNA delivery in *B. tabaci*, the dsRNA was expressed in the host plant by virus‐induced gene silencing (VIGS). As expected, dsRNA expressed in the VIGS‐treated plant hosts was well delivered to the intestine of *B. tabaci* as well as *F. occidentalis* and killed both sucking insects. To facilitate the dsRNA delivery to the plant tissues, two dsRNA formulations were compared in the control efficacy of the sucking insects. Compared to unformulated dsRNA, either chitosan‐ or layered double hydroxide (LDH)‐formulation was effective to enhance the control efficacy against both sucking insects. Furthermore, LDH formulation was much more effective to control *B. tabaci*. When LDH‐formulated dsRNA was sprayed to hot peppers infested by whiteflies and thrips, it resulted in over 80% control efficacy against the whiteflies and the thrips. These control efficacies were similar to those of a chemical insecticide, Spinosad. In addition, compared to the unformulated dsRNA, the LDH‐formulated dsRNA exhibited a relatively long residual control efficacy, which may reduce the spraying amount of the dsRNA insecticide.

**CONCLUSION:**

These results suggest that LDH formulation is useful for dsRNA delivery against the two sucking insects. The LDH‐dsRNA specific to *vATPase‐B* of *B. tabaci* was effective to control both whitefly and thrips infesting hot peppers. © 2025 The Author(s). *Pest Management Science* published by John Wiley & Sons Ltd on behalf of Society of Chemical Industry.

## Graphical abstract

A vacuolar type ATPase subunit B (*vATPase‐B*) gene is identified and expressed in the midgut of *Bemisia tabaci* and *Frankliniella occidentalis*. The double‐stranded RNA (dsRNA) specific to *vATPase‐B* was expressed in hot peppers by virus‐induced gene silencing (VIGS) and killed both whitefly and thrips. Layered double hydroxide (LDH)‐formulated dsRNA sprayed on hot peppers killed thrips and whiteflies with > 80% control efficacy.

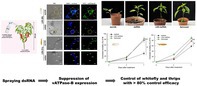

## 1 INTRODUCTION

The whitefly, *Bemisia tabaci* (Gennadius), is a phloem‐feeding insect and causes serious damage against over 600 species of plants including food, vegetable, fiber, and ornamental crops.^1^ It is highly invasive and now occurs in most subtropical and temperate countries over the world.^2^ It is not a single species but species complex consisting of at least 43 cryptic species.^3^ Some of them are notorious for transmitting plant viruses during the feeding process,^4^ in which two cryptic species, Middle East‐Asia Minor 1 (MEAM1 = B biotype) and Mediterranean (MED = Q biotype), are highly invasive and rapidly replace native whitefly species.^5^

The western flower thrips, *Frankliniella occidentalis*, is an economically important invasive pest infesting various horticultural crops and ornamental plants. Since it was first detected in 1993 in a southern island, it has quickly spread to all over the country in Korea.^6^ Even though more than 20 different chemical insecticides have been applied to control this insect pest, its outbreaks give serious yield loss of crops especially those cultivating in glasshouses.^7^ It causes a feeding damage to a high‐value crop, the hot pepper (*Capsicum annuum*), and also transmit a plant virus, tomato spotted wilt virus.^8^

In addition to the thrips, *B. tabaci* infests the hot pepper and transmits plant viruses.^9^, ^10^ The whiteflies transmit tomato yellow leaf curl virus, which is a member of the genus Begomovirus with a single‐stranded DNA genome.^11^ Even though the hot peppers are asymptomatic, they serve as an acquisition source for the viral transmission by *B. tabaci* to tomato.^12^ Thus, these two insect pests should be controlled to increase crop yield. Due to their development of insecticide resistance, a novel control tactic is urgently needed to be developed.^8^, ^11^

To avoid a massive use of chemical insecticides, a biopesticidal double stranded RNA (dsRNA) was commercially registered by the US Environmental Protection Agency (EPA) to control the insect pest, *Leptinotarsa decemlineata*, by a spraying method (https://www.epa.gov/pesticides/epa-registers-novel-pesticide-technology-potato-crops). This triggers various practical research to develop novel dsRNA insecticides to control various insect pests.^13^ The control principle of dsRNA is based on RNA interference (RNAi) specific to a target gene.^14^ Exogenous dsRNA is processed by Dicer to produce small dsRNA fragments, which are further processed to produce small interference RNA (siRNA) by an RNase called Argonaute (Ago) in RNA‐induced silencing complex (RISC).^15^ However, spraying dsRNA is not likely to be effective to control the sucking mouth part insect pests because dsRNA is not well delivered into the internal plant tissues. Alternatively, different dsRNA formulations have been developed to control the sucking insects as well as plant pathogens infecting the internal plant tissues.^16^ Layered double hydroxide (LDH) called Bio‐Clay is among the formulations that have been tested against *B. tabaci* and have shown potential to control the insect pest.^17^

Vacuolar‐type ATPase subunit B (vATPase‐B) is an effective target gene for the RNA interference (RNAi) of *F. occidentalis* and the resulting dsRNA turned out to be effective to control the thrips.^18^, ^19^ Chitosan‐formulation of the dsRNA much improved the control efficacy against *F. occidentalis* when it was sprayed to the hot peppers by protecting dsRNase attacks from the gut lumen and enhancing stability under environmental stresses such as ultraviolet (UV) and high temperatures.^20^ The amino acid sequence of *vATPase‐B* is highly conserved especially among insects^21^ and suggests its common target for the dsRNA to control the thrips and the whitefly. However, these two sucking mouth part insects have different feeding behaviors where *B. tabaci* is a sap‐feeder from phloem while *F. occidentalis* is a mesophyll feeder by scratching the leaf surface by the mandible.^22^ Thus, the sprayed dsRNA can be fed by *F. occidentalis* but not by *B. tabaci*.

This study tested the insecticidal activity of dsRNA specific to *vATPase‐B* against *B. tabaci* based on its toxicity to *F. occidentalis* in order to control both insect pest using a single dsRNA. This study clarified the differential toxicity of the sprayed dsRNA between the two species. To enhance the insecticidal activity of the sprayed dsRNA against *B. tabaci*, this study formulated the dsRNA with LDH and applied to *B. tabaci*. It also assessed the residual effect of LDH‐formulated dsRNA to reduce the sprayed dsRNA amount in field conditions.

## 2 MATERIALS AND METHODS

### 2.1 Insects rearing

The adult whiteflies of *B. tabaci* were collected from the hot peppers cultivated in a glasshouse located in Andong, Korea. Hot peppers (*C. annuum*) of Kwari variety were cultivated under control conditions at 25 ± 1 °C, 12 h:12 h (light/dark) photoperiod, and approximately 60% relative humidity. The whitely colony was maintained on the hot pepper seedlings in laboratory conditions of 25 ± 1 °C, 14 h:10 h (light/dark) photoperiod, and 70 ± 5% relative humidity according to the method described by Kil *et al*.^12^ A laboratory colony of *F. occidentalis* was previously established since collection from a hot pepper field at Andong, Korea and maintained under laboratory conditions. Larvae and adults were fed germinated kidney beans (*Phaseolus coccineus* L.) as their diet. The bean (Kangnam variety) was purchased from Yechon Agricultural cooperation (Yechon, Korea). Newly hatched larvae on the beans were transferred daily to breeding dishes (100 mm × 40 mm; SPL Life Sciences, Pocheon, Korea). All experiments in the hot peppers were conducted using 4‐week‐old plants to ensure uniformity and consistency in developmental stages across the assays.

### 2.2 Bioinformatics

The *vATPase‐B* sequences of *B. tabaci* and *F. occidentalis* used in this study were retrieved from GenBank using the accession numbers MN738157.1 and KP234253.1. The resulting sequences were examined for open reading frame (ORF) using ORFfinder (https://www.ncbi.nlm.nih.gov/orffinder/). Sequence alignment was performed using ClustalW software from MegAlign (version 6.0; DNASTAR, Madison, WI, USA). Sequence homology of two *vATPase‐B* genes derived from *B. tabaci* and *F. occidentalis* was estimated using the BLAST search engine of the National Center for Biotechnology Information (NCBI, http://blast.ncbi.nlm.nih.gov/Blast.cgi). AlphaFold v3 (https://alphafold.ebi.ac.uk/) was used to construct three‐dimensional structures and analyze active binding site of vATPase‐B protein of *B. tabaci* using an option of Mol*3D Viewer.

### 2.3 RNA extraction and cDNA preparation

Total RNAs were extracted from approximately 50 whiteflies or thrips per replicate or a hot pepper leaf using Trizol reagent (Invitrogen, Carlsbad, CA, USA) according to the manufacturer's instructions. The extracted RNA was resuspended in nuclease‐free water, and its concentration was measured with a spectrophotometer (NanoDrop; Thermo Scientific, Wilmington, DE, USA). For complementary DNA (cDNA) synthesis, 60 ng of RNA per reaction was used with RT‐Premix (Intron Biotechnology, Seongnam, Korea) containing an oligo‐dT primer, following the manufacturer's protocol.

### 2.4 Real‐time quantitative polymerase chain reaction (qPCR)

Synthesized cDNAs were used for polymerase chain reaction (PCR) amplification with DNA Taq polymerase (GeneALL, Seoul, Korea) and gene‐specific primers (Supporting Information Table S1) under the following conditions: an initial denaturation at 95 °C for 3 min, followed by 35 cycles of denaturation at 95 °C for 1 min, annealing at 55° for 1 min, and extension at 72 °C for 1 min. The PCR reaction mixture (25 μL) consisted of a DNA template (1 μL), 1 μL of dNTPs (2.5 mm each), 1 μL of each primer (10 pmol), 1 μL of Taq polymerase (2.5 units/μL), and 20 μL of deionized distilled water.

Quantitative PCR (qPCR) was performed using a real‐time PCR instrument (StepOnePlus Real‐Time PCR System, Applied Biosystems, Singapore) and Power SYBR Green PCR Master Mix (Life Technologies, Carlsbad, CA, USA), following the guidelines of Bustin *et al*.^23^ The qPCR reaction mixture (20 μL) included 10 μL of Power SYBR Green PCR Master Mix, 2 μL of cDNA template (60 ng/μL), 1 μL each of forward and reverse primers, and the remaining volume with deionized distilled water. *Elongation factors* (*Bt‐EFα* or *Fo‐EF1*) were used as the reference genes, with their specific primers listed in Table S1. Melting curve analysis was performed to verify the presence of a single specific PCR product. Quantitative analysis was conducted using the comparative CT (2^−∆∆CT^) method,^24^ with three independent replicates for each experiment.

### 2.5 Identification of whitefly species complex

Genomic DNA extraction was performed using QuickExtract DNA Extraction Solution (LGC Biosearch Technologies, Hoddesdon, UK), according to the manufacturer's protocol. Each sample for extraction consisted of a single individual from the same colony. PCR amplification was conducted with Cl‐J‐2195 (5'‐TTGATTTTTTGGTCATCCAGAAGT‐3') and TL2‐N‐3014 (5'‐TCCAATGCACTAATCTGCCATATTA‐3') primers to get approximately 650 bp PCR product matched to cytochrome oxidase I (COI) at the 3' region. The resulting PCR product was bi‐directionally sequenced and aligned to 212 COI sequences^3^ covering 42 species complexes of *B. tabaci* to construct a phylogeny tree using neighbor‐joining (NJ) operating in MEGA 11.0. Bootstrap values were obtained with 1000 repetitions.

### 2.6 Preparation of linear dsRNA

Template DNAs were amplified through PCR using gene‐specific primers (Table S1) with the T7 promoter sequence attached to their 5′ ends, following previously established protocols.^19^, ^20^, ^21^ The manufacturer's instructions subjected the PCR products to *in vitro* transcription using the MEGAscript RNAi Kit (Ambion, Austin, TX, USA). The synthesized dsRNAs were mixed with Metafectene PRO at a 1:1 volume ratio and incubated at room temperature for 30 min to create a dsRNA–liposome complex. RNAi efficiency was evaluated via qPCR 48 h after treatment.

### 2.7 Chitosan formulation

To prepare chitosan–dsRNA, chitosan derived from crab shells with a deacetylation level of ≥ 75% (Sigma‐Aldrich Korea, Seoul, Korea) was dissolved in a 0.1 m sodium acetate solution (a mixture of 0.1 m NaC_2_H_3_O_2_ and 0.1 m acetic acid in deionized water, pH 4.5) and kept at room temperature (RT). A solution containing 50 μL of dsRNA was first combined with 100 μL of 100 mm sodium sulfate solution (prepared by dissolving 100 mm Na_2_SO_4_ in deionized water). This mixture was then added to 100 μL of the chitosan solution, pre‐dissolved in the 0.1 m sodium acetate solution. The resulting mixture was heated to 55 °C for 1 min and vortexed for 30 s to promote the formation of nanoparticles. The mixture was then centrifuged at 14 000 × *g* for 10 min at RT, producing a pellet that was washed three times with deionized water and resuspended in MilliQ ultrapure water. Finally, the nanoparticles were sonicated for 5 min at 25 °C using an ultrasonic liquid processor (Powersonic 405, Hwashin, Shanghai, China) before being used in subsequent experiments. The chitosan‐formulation resulted in 500 μg dsRNA/mL.

### 2.8 LDH formulation

To produce dsRNA‐loaded LDH–dsRNA, two separate solutions were initially prepared: one containing 75 mm magnesium nitrate (Mg(NO_3_)_2_) and the other containing 25 mm aluminum nitrate (Al(NO_3_)_3_). These solutions were gradually introduced into 1 m sodium hydroxide under continuous stirring. The resulting mixture was stirred and heated for 4 h. After then, the mixture was centrifuged, and the resulting precipitate was washed three times with distilled deionized water. The precipitate was then autoclaved for 30 min and dried at 100 °C for another 30 min to produce LDH, which was then mixed with dsRNA in a 1:1 volume ratio (*v/v*) and vortexed to ensure uniform mixing. The dsRNA–LDH complex was subsequently centrifuged and washed three times with double‐distilled water (ddH_2_O) to remove any excess components, yielding the final dsRNA‐loaded LDH product. The LDH formulation resulted in 250 μg dsRNA/mL.

### 2.9 Virus‐induced gene silencing (VIGS)

Virus‐induced gene silencing (VIGS) technique was used to produce dsRNA specific to *vATPase‐B* in the hot peppers according to the method described by Zhang and Thomma^25^ with slight modifications. A partial sequence of *vATPase‐B* of *B. tabaci* was amplified with gene‐specific primers (Table S1) and cloned into the pCR2.1‐TOPO vector using the TOPO TA Cloning Kit (Invitrogen). After transformation to *Escherichia coli* Top ten, the transformed clones were cultured in Luria–Bertani (LB) medium supplemented with ampicillin, and the multiplied plasmid DNA was extracted using the Expin^TM^ Plasmid SV Mini Kit (GeneALL). The cloned plasmids were confirmed through sequencing by Macrogen (Seoul, Korea) and subsequently digested with BamHI and XbaI (Takara Korea, Seoul, Korea). The digested fragments were ligated into the pTRV2 vector to create pTRV2‐*vATPase B*.

The resulting pTRV2‐*vATPase‐B* vector was transformed into *E. coli* and grown overnight on LB agar plates containing kanamycin (100 μg/mL) at 37 °C. Verified recombinant plasmids (10 μL) were then introduced into *Agrobacterium tumefaciens* via electroporation. The transformed *A. tumefaciens* was cultured on LB agar supplemented with kanamycin and rifampicin, and incubated for 72 h at 28 °C. Positive colonies were cultured in 50 mL of LB broth containing kanamycin and rifampicin. The cells were pelleted by centrifugation, resuspended in an infiltration buffer (10 mm MES, 10 mm magnesium chloride (MgCl_2_), and 200 μm acetosyringone), and adjusted to an optical density measured at 600 nm (OD_600_) of 1.3–1.5. The suspension was allowed to incubate at RT for 2–3 h before use.

For plant inoculation, two *A. tumefaciens* clones containing either of pTRV1 or pTRV2‐*vATPase‐B* were mixed in a 1:1 volume ratio. Small wounds were made on the abaxial side of the cotyledons of hot pepper seedlings using a needle. A 1 mL syringe without a needle was used to inject the bacterial mixture into the wounded area. After infiltration, the seedlings were maintained in a growth chamber at 25 ± 1 °C.

### 2.10 Fluorescence *in situ* hybridization (FISH) assay

Individual whitefly and thrips adults were dissected to isolate the entire intestine. The dissected tissues were mounted on sterilized glass slides and fixed with 4% paraformaldehyde for 1 h at RT. The tissues were then washed with 1× phosphate‐buffered saline (PBS) and permeabilized with 1% Triton X‐100 in PBS for 2 h at RT. After another PBS wash, the gut tissues were incubated in 2× saline‐sodium citrate (SSC) solution containing 25 μL of pre‐hybridization buffer (2 μL yeast tRNA, 2.5 μL 20× SSC, 4 μL 50% dextran sulfate, 2.5 μL 10% sodium dodecyl sulfate, and 14 μL deionized water) for 1 h in a dark, humid chamber at 42 °C.

The pre‐hybridization buffer was then replaced with the hybridization buffer comprising 5 μL of deionized formamide, 1 μL of fluorescein‐labeled oligonucleotide, and 19 μL of pre‐hybridization buffer. DNA oligonucleotide probes designed for *vATPase‐B* gene were labeled with fluorescein amidite (FAM) at the 5′ end and purified through high‐performance liquid chromatography (Bioneer, Daejeon, Korea). These probes included an antisense sequence complementary to the target messenger RNA (mRNA) and a sense probe used as a negative control.

The slides were covered with RNase‐free coverslips and incubated at 42 °C in a humid chamber for 16–17 h to allow hybridization. Post‐hybridization, the tissues were washed twice with 4× SSC for 10 min each and treated with 4× SSC containing 1% Triton X‐100 at RT for 5 min. This was followed by three additional washes with 4× SSC. The midguts were then incubated at 37 °C in the dark for 30 min with 1% anti‐rabbit‐FITC (fluorescein isothiocyanate) conjugated antibody (Thermo Scientific) diluted in PBS. After antibody treatment, the tissues were washed twice with 4× SSC for 10 min each and once with 2× SSC. The samples were then air‐dried, a drop of 50% glycerol was added, and they were incubated for 15 min at RT. Coverslips were placed on the samples, which were subsequently analyzed using a fluorescence microscope (DM2500, Leica, Wetzlar, Germany) at 200× magnification.

### 2.11 Insecticidal bioassay of dsRNA in laboratory

For the thrips bioassay, beans were immersed in a dsRNA solution for 20 min. After drying, the treated beans were placed in breeding dishes and fed to ten *F. occidentalis* individuals per replication. For the whitefly bioassay, a similar dipping method was used, in which hot pepper leaves were used in place of beans. Each treatment was performed in triplicate. As a control, a partial sequence of an enhanced green fluorescent protein (*EGFP*) gene was used to synthesize control dsRNA (dsCON). Mortality was scored 7 days after 24‐h feeding.

### 2.12 Insecticidal bioassay of dsRNA in hot pepper pot

Each pot (12 cm × 12 cm) had a hot pepper (~15 cm height). Test pots were set in four rows apart by 40 cm, in which each pot in a row was apart by 50 cm. Each hot pepper was infested with 30–40 whiteflies or thrips before the dsRNA treatment. The pots were arranged in a randomized block design with three blocks for replication. Each plant received 7.5 mL of dsRNA spray at a concentration of 250 ppm. The control group was treated with the same volume of dsCON.

### 2.13 Control efficacy test of dsRNA in glasshouse cultivating hot peppers

The glasshouse assay consisted of three rows of young hot pepper plants, each spaced 50 cm apart, with individual plants positioned 40 cm apart. The plants, approximately 25 cm in height, were treated as individual experimental units. Each hot pepper was initially infested with approximately 80 whiteflies or thrips before the dsRNA application. The treatments were arranged in a randomized block design with three blocks for replication. Each plant was sprayed with 15 mL of a solution containing 250 μg/mL of either LDH‐formulated dsRNA or unformulated dsRNA. Water was applied as untreated negative control and the resulting natural mortality was used to calculate the control efficacies of the treatments. Alternatively, a commercial Spinosad formulation (Excellent, Dongbang Agro, Seoul, Korea) was used at the recommended dose (500 ppm) as a positive control.

### 2.14 Statistical analysis

All analyses were performed using one‐way analysis of variance (ANOVA) with the PROC GLM procedure in the SAS program.^26^ Corrected mortality data were subjected to arcsine transformation before ANOVA. Mean comparisons were conducted using the least significant difference (LSD) test. The study included three independent biological replicates, and results were expressed as the mean ± standard error, generated with SigmaPlot v10.0. For glasshouse experiments, ANOVA was carried out based on a randomized block design, with mean separation conducted using Tukey's honestly significant difference (HSD) test.

## 3 RESULTS

### 3.1 Identification of *B. tabaci* in species complex

An isolate of *B. tabaci* was identified with its COI sequence (Supporting Information Fig. S1(A)). The sequence was aligned with other known cryptic species of *B. tabaci* and showed that our isolate was clustered with Mediterranean type (MED) among 42 types used in this analysis (Fig. S1(B)).

### 3.2 Expression profile of *vATPase‐B* in *B. tabaci*

**Figure 1:**
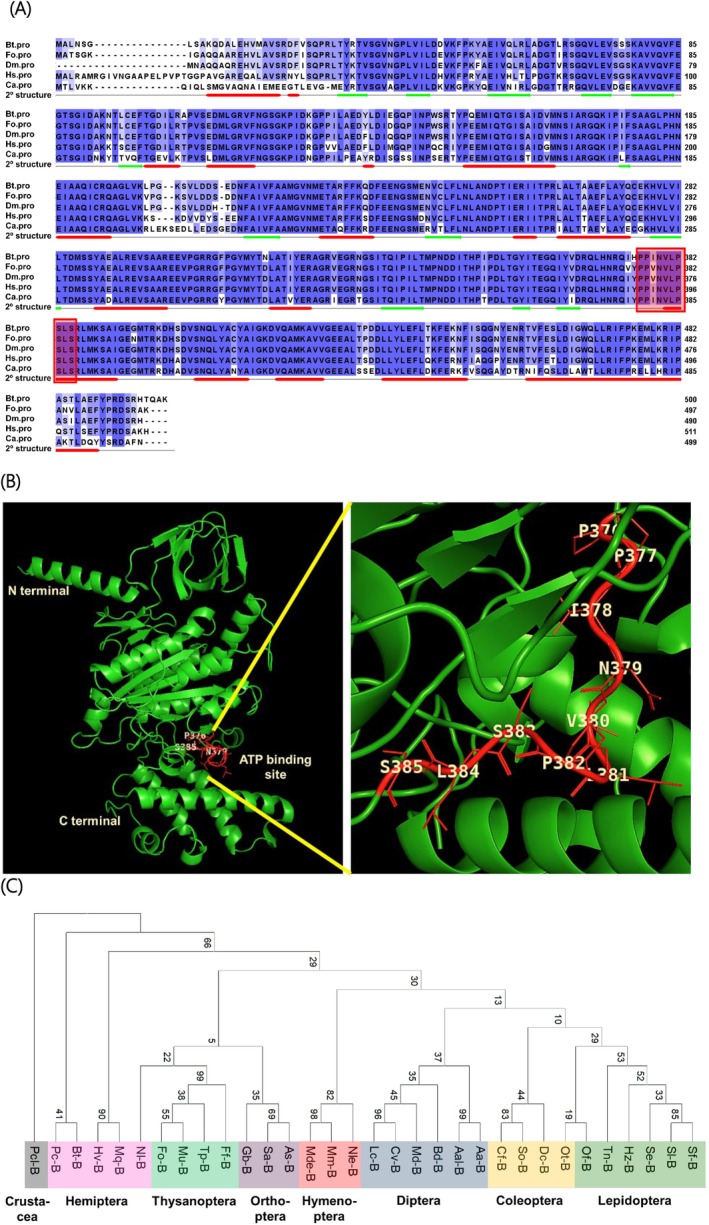
Prediction of *vATPase‐B* gene (GenBank accession number: XP_018896879.1) in *Bemisia tabaci* (‘Bt’) genome. (A) Its predicted amino acid sequence and alignment with other *vATPase‐B* sequences of *Frankliniella occidentalis* (‘Fo’, KAE8747108.1), *Drosophila melanogaster* (‘Dm’, NP_001163597.1), *Homo sapiens* (‘Hs’, NP_001684.2), and *Capsicum annuum* (‘Ca’, XP_016545148.2). Conserved amino acid sequences are denoted in blue‐colored boxes. The secondary (2°) structure predicted from *B. tabaci* sequence shows 17 α‐helix (red‐colored bars) and 13 β‐barrel (green‐colored bars) structures. The adenosine triphosphate (ATP)‐binding site is denoted by a red‐colored square box. (B) The active sites of the *vATPase‐B* gene in *B. tabaci* in a three‐dimensional structure constructed by AlphaFold v3. Ten amino acid residues (P376‐S385) were identified in the ATP‐binding site. (C) Phylogenetic analysis of insect *vATPase‐B* genes from different insect orders. The phylogenetic tree was generated by the NJ method using MEGA6.0. Bootstrapping values were obtained with 1000 repetitions to support branch and clustering. Amino acid sequences were retrieved from GenBank with the accession numbers shown in Supporting Information Table S2.

**Figure 2:**
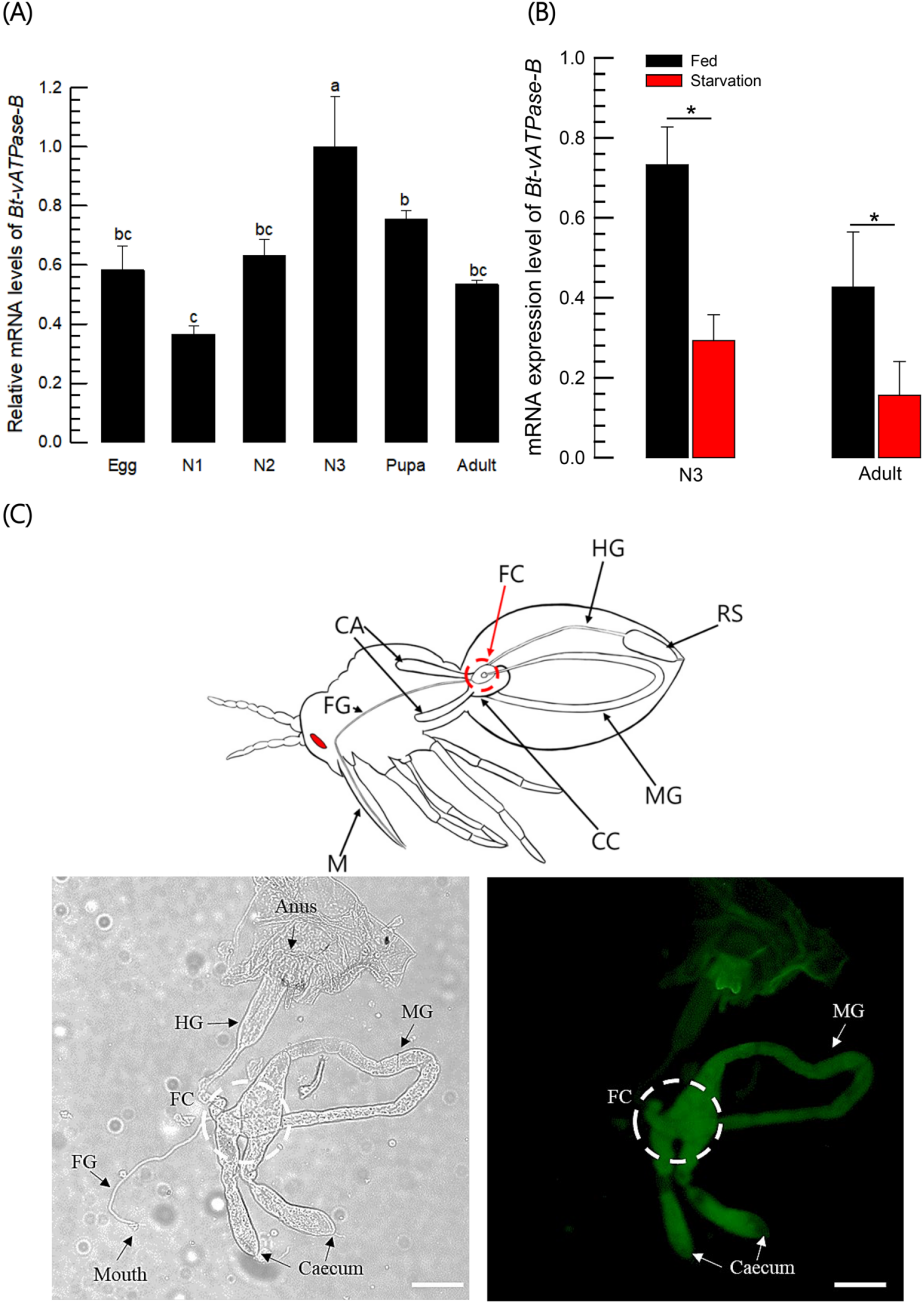
Expression profile of *vATPase‐B* gene (*Bt‐vATPase‐B*) in *Bemisia tabaci*. (A) Its expression pattern in different developmental stages of *B. tabaci*. Each replication used ~200 eggs, 50 individuals of each three nymphal stages (N1–N3), 50 pupae, and 50 adults for RNA extraction. Each treatment was replicated three times. (B) Influence of feeding activity on the expression of *Bt‐vATPase‐B*. Two developmental stages were starved for treatment for 24 h, which was compared with the feeding control at the starvation period. Expression level of a constitutively expressed gene, *EF‐1*, was used to normalize the expression levels of *Bt‐vATPase‐B*. Three replications were used per treatment. Different letters or asterisks above standard deviation bars indicate significant differences among means at Type I error = 0.05 (LSD test). (C) Expression analysis of *vATPase‐B* in the intestine of *B. tabaci* using FISH. A diagram indicates the different parts of the intestine of *B. tabaci* showing different parts of the digestive system: mouth (‘M’), foregut (‘FG’), filter chamber (‘FC’), caecum (‘CA’), connecting chamber (‘CC’), midgut (‘MG’), hindgut (‘HG’) and rectal sac (‘RS’). The intestine was observed under a differential interference contrast (left panel) mode at 100× magnification. The scale bar represents 0.1 mm. *Bt‐vATPase‐B* expression (green, right panel) was detected with an antisense probe. To confirm the specificity, sense probe was used and did not detect any part. A fluorescent microscope (DM2500) was used to observe the samples in a FITC fluorescence mode.

An ORF (500 amino acid residues) of *vATPase‐B* was predicted from a genome of *B. tabaci* and showed ATPase catalytic site (Fig. 1(A),(B)). It was aligned with other known insect vATPase‐B proteins and showed that it is clustered with other hemipteran orthologs and closely related with *F. occidentalis* in Thysanoptera (Fig. 1(C)). It was expressed in eggs and other developmental stages, in which the highest expression was observed at the last nymphal stage (Fig. 2(A)). However, its expression levels were modulated by feeding behavior. Starvation significantly suppressed its expression levels in both nymphal and adult stages (Fig. 2(B)). This led us to examine its expression in the intestine of *B. tabaci* to predict its physiological function in digestion and absorption. Adult whitefly was dissected and showed foregut, midgut, and hindgut, in which the midgut was subdivided into two caeca and ascending and descending midgut (Fig. 2(C), left panel). Interestingly, the *vATPase‐B* was highly expressed in the filter chamber along with midgut from fluorescence *in situ* hybridization (FISH) analysis (Fig. 2(C), right panel).

### 3.3 Spraying the unformulated dsRNA was toxic to *F. occidentalis* but not to *B. tabaci*

**Figure 3:**
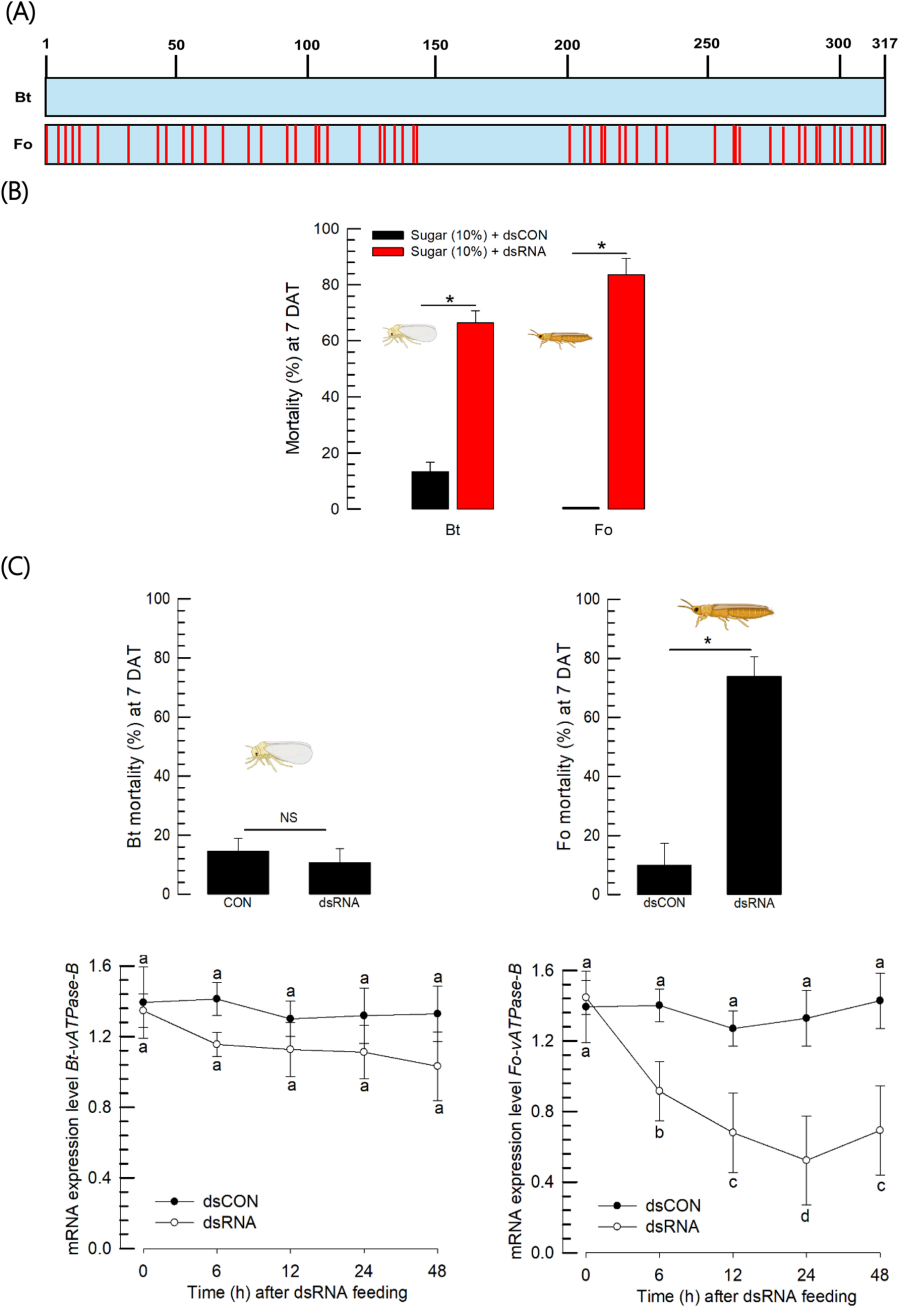
Influence of dsRNA specific to *vATPase‐B* gene (*Bt‐vATPase‐B*) of *Bemisia tabaci* on survival of *B. tabaci* (‘Bt’) and *Frankliniella occidentalis* (‘Fo’). (A) Sequence comparison of a partial gene (317 bp) of *Bt‐vATPase‐B* used for constructing dsRNA with the corresponding gene of *F. occidentalis*. Blue region indicates identical sequences while red lines represent different sequences. These partial sequences exhibit 85.7% homology and contain 19 loci with at least a 16‐bp consecutive identical sequence^34^ match. (B) Toxicity test of the dsRNA against the two insect species using sugar‐feeding assay. The dsRNA was mixed with 10% sugar solution into a final concentration of 500 ppm and fed to test insects, which had been starved for 6 h. (C) Toxicity test of the dsRNA against the two insect species using diet‐dipping assay. Test dsRNA suspension (500 ppm) in distilled water was delivered orally by providing dsRNA‐soaked hot pepper leaves (whitefly) and beans (thrips). Mortality (%) was assessed seven days after treatment (‘DAT’). Each treatment consisted of ten adults and was replicated three times. The control (‘dsCON’) treatment used dsRNA specific to *EGFP* gene. Asterisk or different letters above the standard deviation bars indicate significant differences among means at Type I error = 0.05 (LSD test). ‘NS’ stands for no significant difference. RNAi effects were assessed by qPCR, in which expression levels were normalized using expression levels of elongation factors: *Bt‐EF1* for *B. tabaci* and *Fo‐EF1* for *F. occidentalis*. Each treatment was performed with three replicates.

Two *vATPase‐B* genes of *B. tabaci* and *F. occidentalis* are highly homologous in ORF sequences (Fig. S2). Based on this high homology, we constructed 317 bp‐long dsRNA specific to *vATPase‐B* of *B. tabaci*, in which two sequences showed 85.7% homology and shared several consecutively identical fragments longer than 16‐mer (Fig. 3(A)).

The constructed dsRNA was fed to test insects using sugar solution (Fig. 3(B)). Both insects of *B. tabaci* and *F. occidentalis* suffered from the dsRNA and showed high mortalities. However, when the dsRNA was fed by a leaf‐dipping method, its toxicity was only observed in *F. occidentalis* but not in *B. tabaci* (Fig. 3(C), upper panel). RNAi efficiency was analyzed in the test insects after the dsRNA feeding (Fig. 3(C), lower panel). As expected, the target gene expression was highly reduced in *F. occidentalis* but not in *B. tabaci*.

### 3.4 Hot peppers expressing dsRNA using VIGS were toxic to both *F. occidentalis* and *B. tabaci*

**Figure 4:**
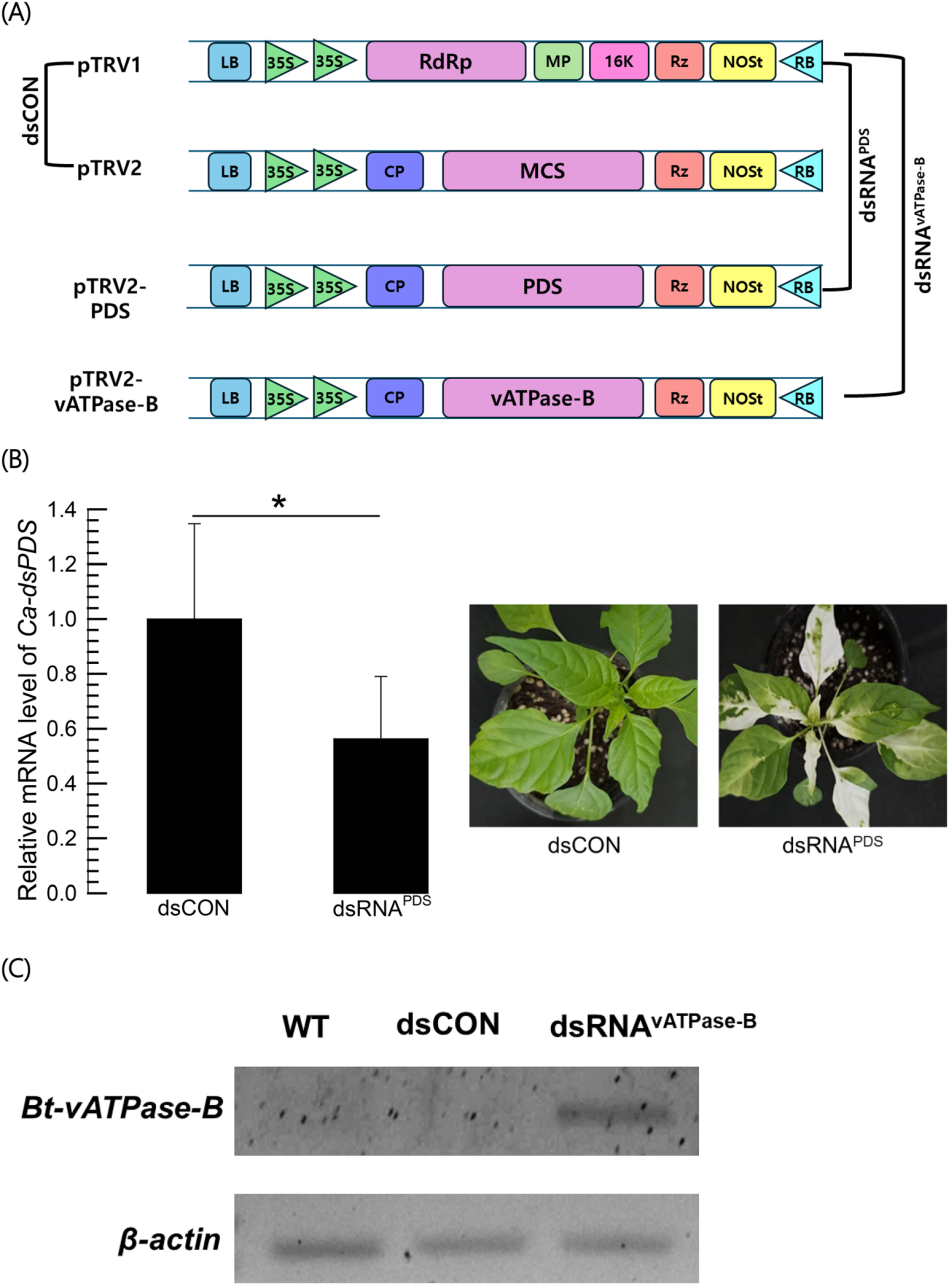
Expression of dsRNA specific to *vATPase‐B* (*Bt‐vATPase‐B*) of *Bemisia tabaci* in a hot pepper (*Capsicum annuum*: Ca) using a VIGS technique. (A) VIGS components. VIGS was performed by co‐injection of pTRV1 and pTRV2 vectors. The multiple cloning site (MCS) in pTRV2 was used to clone a partial *Bt‐vATPase‐B* (vATPase‐B) gene (317 bp) to produce dsRNA^vATPase‐B^ or a partial *phytoene desaturase* (PDS) gene (324 bp) to produce dsRNA^PDS^. (B) RNAi efficiency using VIGS against *Ca‐PDS* expression in hot peppers. After 32 days post‐injection, *Ca‐PDS* mRNA expression levels in control and treatment were assessed by qPCR, in which *Ca‐β‐actin* was used to normalize the expression level in each sample. The asterisk above the standard deviation bars indicates significant differences between control and treatment based on LSD test at a Type I error rate of 0.05. Photographs represent the RNAi efficacy by decolorization on the leaves. (C) Insect dsRNA production in hot peppers assessed by PCR. Wild‐type (‘WT’) stands for hot peppers without any VIGS injection. A negative control (‘dsCON’) used an empty pTVR2 without any insertion to MCS.

For VIGS, a recombinant expression vector was constructed using pTRV2 (Fig. 4(A)). To test the VIGS system, a recombinant pTRV2 expressing dsRNA specific to phytoene desaturase (*PDS*) gene was infected to young hot peppers along with pTRV1. The target gene expression levels were significantly reduced (Fig. 4(B)). The treated hot peppers showed white‐colored leaves probably by blocking carotenoid biosynthesis due to lack of PDS.

**Figure 5:**
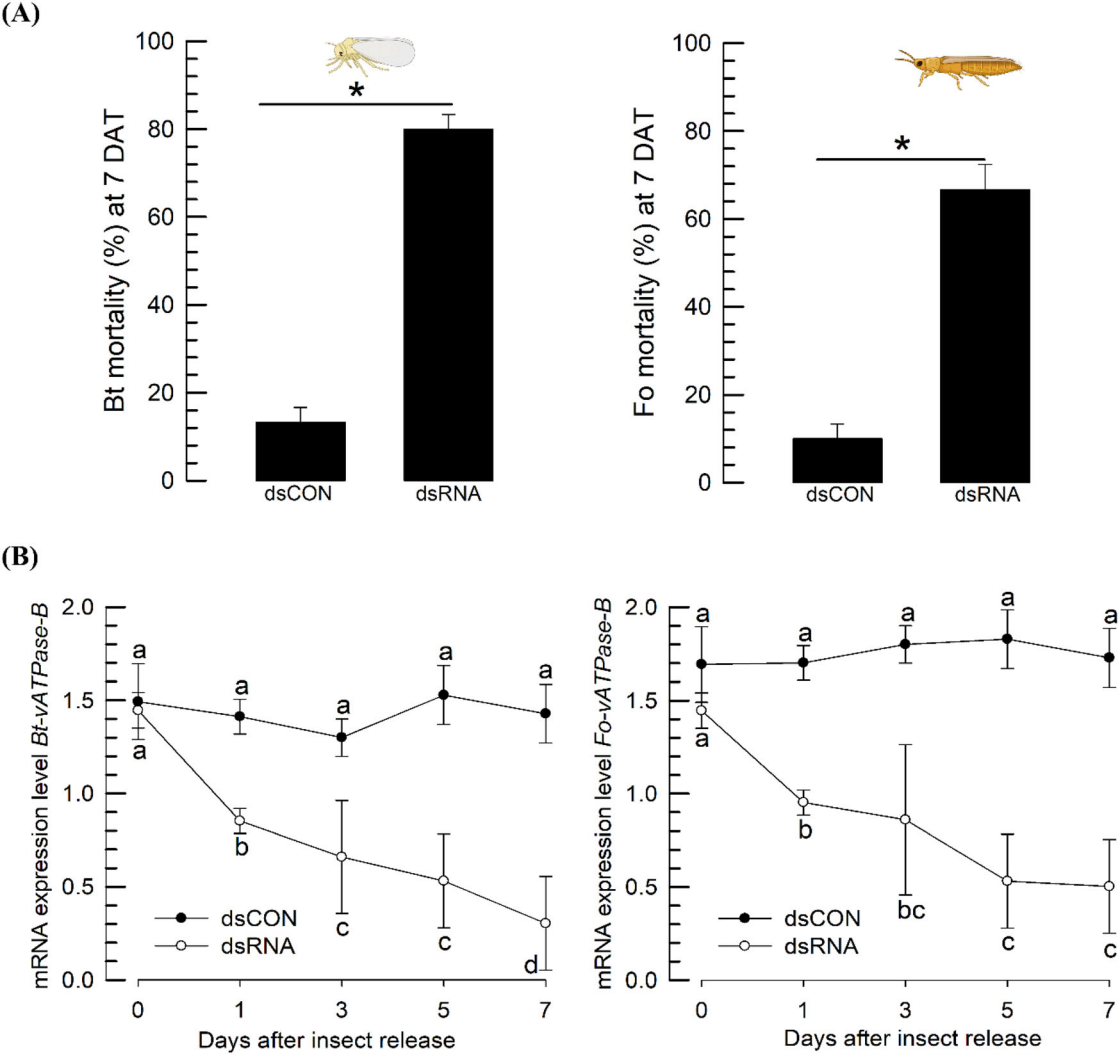
Insecticidal activity of dsRNA expressed in hot peppers using VIGS treatment. (A) Insecticidal activity of dsRNA specific to *vATPase‐B* (*Bt‐vATPase‐B*) of *Bemisia tabaci* expressed in the hot peppers against *B. tabaci* (‘Bt’) and *Frankliniella occidentalis* (‘Fo’). Mortality (%) was assessed 7 days after treatment (‘DAT’). Each treatment consisted of ten adults and was replicated three times. The control (‘dsCON’) treatment represents the injection of the non‐recombinant VIGS treatment. The asterisk above the standard deviation bars indicates significant difference between control and treatment means at a Type I error rate of 0.05 (LSD test). (B) RNAi effects were assessed by qPCR, in which expression levels were normalized using the expression levels of elongation factors: *Bt‐EF1* for *B. tabaci* and *Fo‐EF1* for *F. occidentalis*. Each treatment was performed with three replicates. Different letters above or below standard deviation bars indicate significant difference among means at Type I error = 0.05.

The confirmed VIGS technique was used to express a recombinant pTRV2 expressing dsRNA specific to *vATPase‐B* of *B. tabaci* (Fig. 4(C)). In dsCON treatment as a control, *vATPase‐B* mRNA was not detected. In contrast, the recombinant pTRV2 expressed dsRNA specific to *vATPase‐B* in the hot peppers. Then, the whiteflies and the thrips were applied to the hot peppers (Fig. 5(A)). Both test insects suffered from high mortalities in the hot peppers expressing dsRNA specific to *vATPase‐B* whereas they did not show the significant mortalities in the control hot peppers. As expected, the target gene expression was highly reduced in *B. tabaci* as well as in *F. occidentalis* (Fig. 5(B)).

### 3.5 Spraying LDH‐formulated dsRNA was toxic to *F. occidentalis* as well as to *B. tabaci*

**Figure 6:**
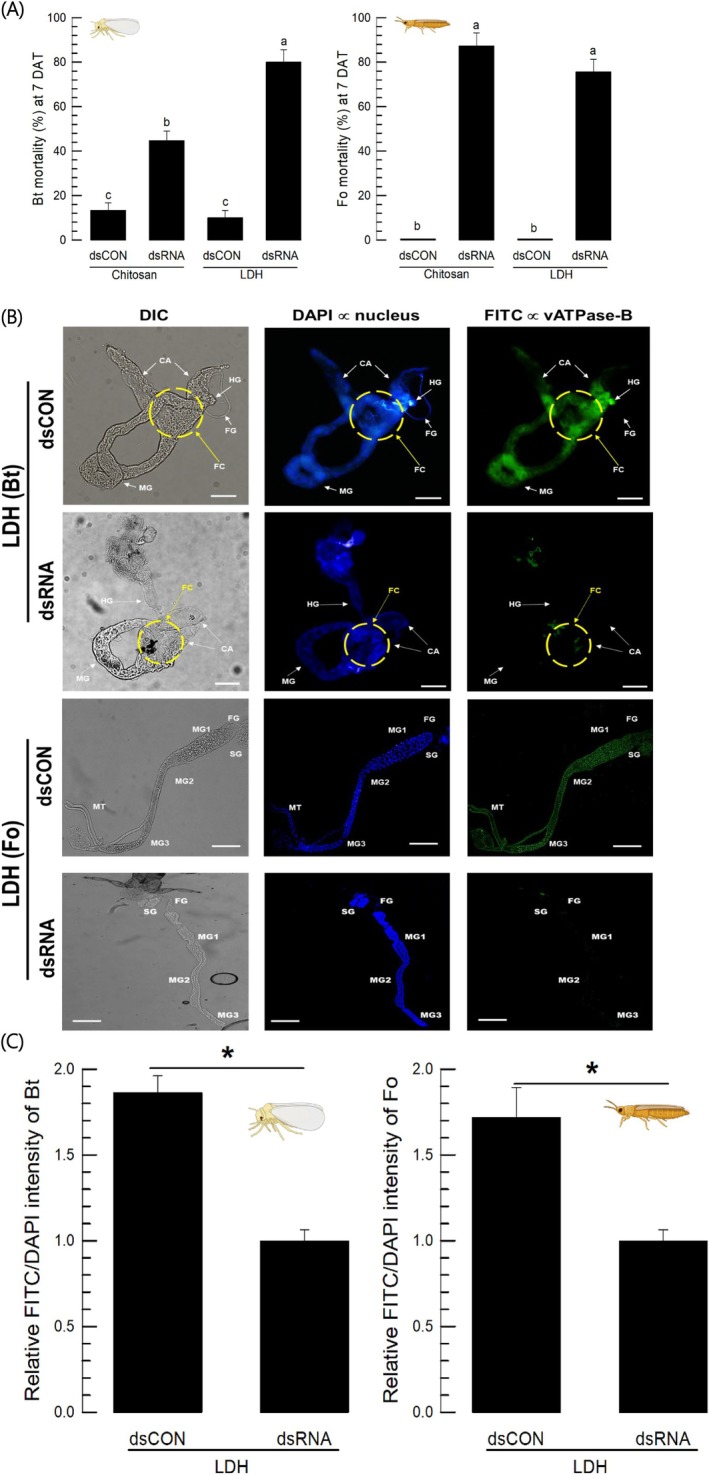
Effect of dsRNA formulation on insecticidal activities against two sucking insects: *Bemisia tabaci* (‘Bt’) and *Frankliniella occidentalis* (‘Fo’). The dsRNA was prepared using a template of *vATPase‐B* (*Bt‐vATPase‐B*) of *B. tabaci*. The dsRNA was formulated with chitosan or LDH in a final concentration of 250 ppm. The control (‘dsCON’) treatment used the formulated dsRNA specific to *EGFP* gene. (A) Diet‐dipping assay. Test dsRNA suspension (250 ppm) in each formulation was delivered orally by providing dsRNA‐soaked hot pepper leaves (whitefly) and beans (thrips). Mortality (%) was assessed 7 days after treatment (‘DAT’). Each treatment used ten adults and was replicated three times. Different letters above the standard deviation bars indicate significant differences among means at a Type I error rate of 0.05 (LSD test). (B) Suppression of *vATPase‐B* expression in the intestines of whitefly and thrips using FISH analysis. After 24 h of dsRNA feeding, the gut was dissected to observe *vATPase‐B* mRNA (green) using antisense probe. Sense probe did not detect any signal (data not shown). A fluorescent microscope (DM2500) was used to view the samples in fluorescence (‘FITC’ against *vATPase‐B*) and differential interference contrast (‘DIC’) modes at 100× magnification. The scale bar represents 0.1 mm. The intestinal parts include foregut (‘FG’), filter chamber (FC’), caecum (‘CA’), midgut (‘MG’), hindgut (‘HG’), salivary gland (‘SG’), and Malpighian tubule (‘MT’). (C) Quantitative analysis of the relative *vATPase‐B* expression levels using FITC signal intensity normalized by DAPI signal intensity for whiteflies and thrips using ImageJ program (https://imagej.net/ij/). Each treatment was independently replicated three times. Asterisk above the standard deviation represents significant difference between control and treatment means at Type I error = 0.05 (LSD test).

To facilitate the dsRNA delivery to the whiteflies, the dsRNA was formulated with chitosan or LDH (Fig. S3). The formulations significantly increased the toxic effect of dsRNA against *B. tabaci*, in which dsRNA formulated with LDH was more efficient in killing the whiteflies compared to that of the chitosan formulation (Fig. 6(A)). However, there was no significant difference in the toxicity between two formulations against *F. occidentalis*.

The whiteflies and the thrips treated with the LDH‐formulated dsRNA showed a significant reduction in the target gene expression levels. In *B. tabaci*, the *vATPase‐B* expression levels were visualized by FISH (Fig. 6(B)). Compared to control, the whiteflies treated with the LDH‐formulated dsRNA showed the loss of the gene expression especially in the filter chamber and midgut (Fig. 6(C)). The expression levels of the target gene in both insects were calculated from the intensity in the intestine. The expression levels were significantly reduced in both insects by the LDH‐formulated dsRNA treatments.

### 3.6 Field application of LDH‐formulated dsRNA to hot peppers

**Figure 7:**
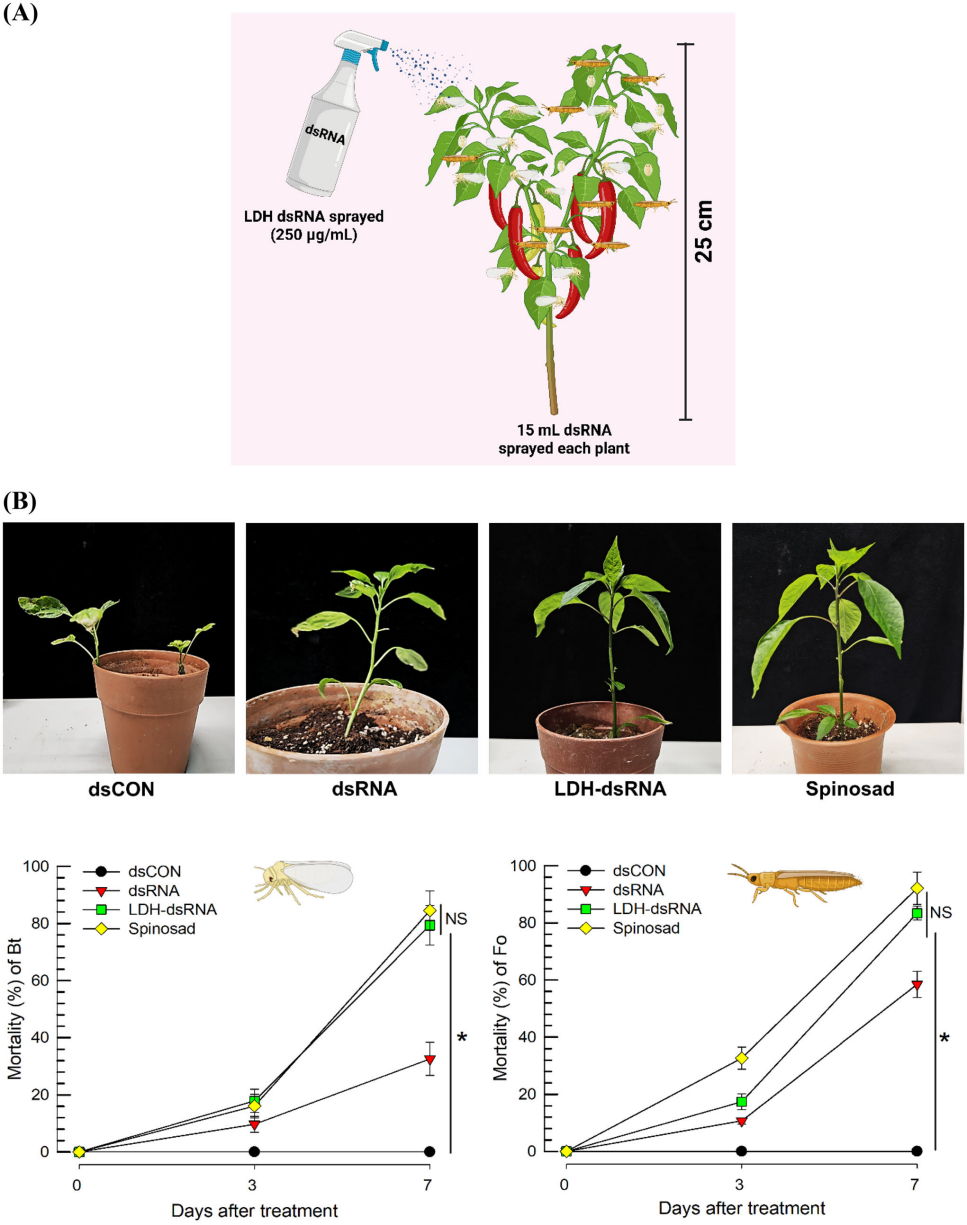
Glasshouse test of LDH‐formulated dsRNA specific to *vATPase‐B* (*Bt‐vATPase‐B*) of *Bemisia tabaci* against *B. tabaci* (‘Bt’) and *Frankliniella occidentalis* (‘Fo’) co‐infesting hot peppers. (A) A schematic diagram illustrating the application of dsRNA to hot peppers. (B) Control efficacies at 3 and 7 days after spray. Photographs represent the hot peppers 21 days after the spray. The ‘dsRNA’ treatment stands for unformulated dsRNA. For positive control, a commercial insecticide (Excellent) with an active ingredient of Spinosad was sprayed at 500 ppm. The initial populations of *B. tabaci* and *F. occidentalis* before spray were over 80 insects of each species per hot pepper. Each plant was an experimental unit and replicated three times in a randomized block design. Significant differences marked asterisks among treatment means were determined using the LSD test with a Type I error rate of 0.05. ‘NS’ represents no significant difference.

Relatively young hot peppers (~25‐cm height) infested with whiteflies and thrips were treated with 250 ppm of LDH‐formulated dsRNA suspension (Fig. 7(A)). In *F. occidentalis*, LDH‐formulated dsRNA compared to unformulated dsRNA increased the control efficacy from 58% to 78% (Fig. 7(B)). In *B. tabaci*, LDH formulation much increased the control efficacy from 23% in the unformulated dsRNA to 82%. In both insects, LDH‐formulated dsRNA had high control efficacy similar to those of a chemical insecticide, Spinosad, treatment. The inset photographs showed the high control efficacy of LDH‐formulated dsRNA, in which two sucking insects co‐infested the hot peppers at the same leaves, in which the thrips were localized on the upper side while the whiteflies were on the lower side.

### 3.7 Long‐lasting control efficacy of LDH‐formulated dsRNA

**Figure 8:**
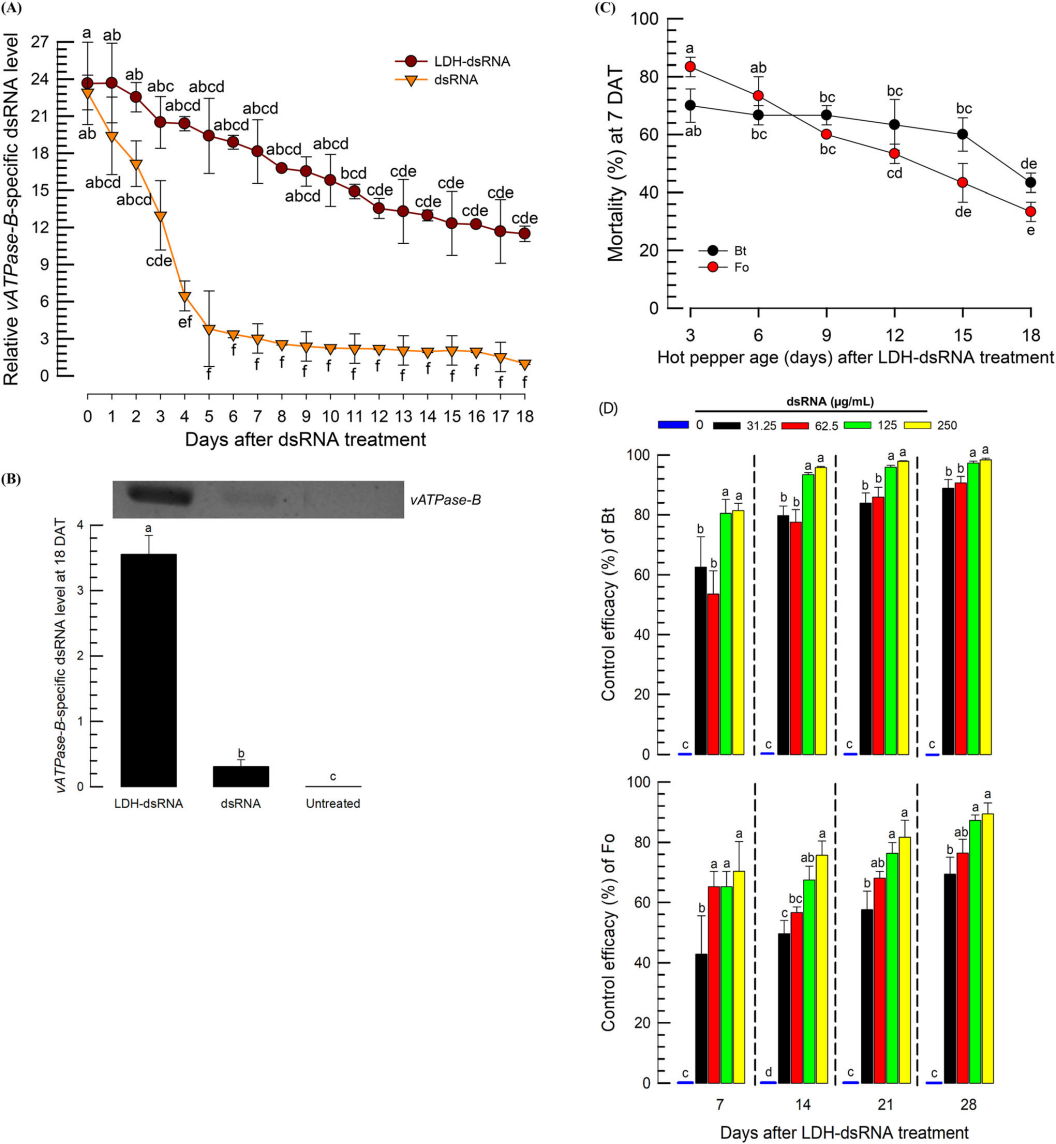
Duration of control efficacy of LDH‐formulated dsRNA specific to *vATPase‐B* (*Bt‐vATPase‐B*) of *Bemisia tabaci* against *B. tabaci* (‘Bt’) and *Frankliniella occidentalis* (‘Fo’) after spraying hot peppers in a glasshouse. (A) Relatively slow decay of the LDH‐formulated dsRNA compared to unformulated dsRNA on hot pepper leaves. The dsRNAs were sprayed on hot peppers at 250 ppm. Then, leaves were collected daily and assessed in the residual dsRNA using qPCR. (B) Residual amounts of the dsRNA in the hot pepper leaves collected on 18 days after treatment (‘DAT’). Note a slight dsRNA residual amount even in unformulated treatment compared to the control (‘untreated’). Expression levels were normalized using *Ca‐β‐actin* gene. Each treatment was performed with three replicates. Gel picture illustrates the relative amounts of the dsRNA specific to *vATPase‐B* using PCR. (C) Control efficacy of the residual dsRNAs in the hot peppers sprayed with LDH‐dsRNA against two sucking insects. (D) Long‐term control efficacy of the LDH‐formulated dsRNA against two sucking insects at different spraying concentrations. Each experimental unit was a hot pepper plant infested with approximately 70–90 whiteflies and 50–60 thrips and replicated three times. Significant differences marked different letters among treatment means using LSD test with a Type I error rate of 0.05.

Persistence and efficacy of LDH‐formulated dsRNA were evaluated by monitoring relative *vATPase‐B* dsRNA levels over 18 days (Fig. 8). Application of unformulated dsRNA led to a rapid decrease in *vATPase‐B* dsRNA level with significant level only for 3 days after application and then minimal levels during the rest of the period (Fig. 8(A)). In contrast, plants treated with LDH‐formulated dsRNA exhibited consistently higher *vATPase‐B* dsRNA level throughout the 18‐day period, indicating slower dsRNA degradation though the dsRNA reduction over time was statistically significant (*F* = 8.87; df = 11, 24; *P* < 0.0001). At 18 days after the spray, the dsRNA specific to *vATPase‐B* remained at a significantly higher level in plants treated with LDH‐formulated dsRNA compared to those treated with unformulated dsRNA or untreated (Fig. 8(B)). It is noticeable that a slight significant level of the dsRNA was detected at 18 days after the treatment in the unformulated dsRNA treatment compared to the control (‘untreated’). The residual dsRNA persistence at the formulated dsRNA treatment was further assessed by treating insects with leaves collected at different intervals following spray application (Fig. 8(C)). Mortality declined over time in both *B. tabaci* and *F. occidentalis*, in which a more pronounced decrease was observed in *F. occidentalis* with a positive correlation (*r* = 0.7112; *P* = 0.0009) between the dsRNA amount and the insecticidal activity.

The long‐lasting control efficacy of the formulated dsRNA suggested a possibility to reduce the spraying amount of the dsRNA to control the insect pests. To test this hypothesis, the formulated dsRNAs at lower amounts were sprayed and analyzed in the dose‐mortality over 4 weeks (Fig. 8(D)). As expected, the control efficacy increased over the time after the dsRNA spray in both sucking insects. The increase of the control efficacy appeared to be more pronounced at the lower spray concentrations (31.25 and 62.5 μg/mL) than the higher concentrations (125 and 250 μg/mL). In *B. tabaci*, all the test concentrations showed over 80% control efficacies though there were slight higher control efficacies at the higher doses. In *F. occidentalis*, the lowest dose (31.25 μg/mL) showed much less control efficacy at 7 days after the treatment by over 20% difference compared to three higher doses. However, at 28 days after the treatment, the lowest dose showed as much similar control efficacy as the higher dose (62.5 μg/mL).

## 4 DISCUSSION

*Bemisia tabaci* is a species complex consisting of at least 43 cryptic species.^3^ Our isolate used in this study was diagnosed to be MED type, which used to be called Q biotype. In Korea, three cryptic species of MED, MEAM1, and JPL are distributed, in which MED has recently invaded the crops and displaced MEAM1 (=B biotype).^27^ This is known in other local populations in Israel, where Q biotype replaced B biotype under exposure to pyriproxyfen while B biotype is more competitive than Q biotype without any insecticide stress.^28^ In Korea, pyriproxyfen and neonicotinoids have been sprayed to control the insect pest in various crops since its invasion in 1998.^29^

In the gut of *B. tabaci*, *vATPase‐B* was expressed in the filter chamber and midgut from FISH analysis in our current study. The vATPase plays a crucial role in the absorption of the digested nutrients in the insect midgut.^30^ It transports proton to the lumen and generates its concentration gradient, which drives an antiporter of potassium ion (K^+^) to keep a high level of the ion concentration in the lumen. The high trans apical voltage across the brush border membrane of the midgut cells drives K^+^‐dependent transport of nutrients.^31^ It consists of two functional domains: transmembrane domain (V0) and cytosolic domain (V1). V1 domain consists of eight subunits (A–H), in which B subunits form a catalytic site for ATPase. Filter chamber functions in osmoregulation by bypassing excess water, ions, and soluble carbohydrates from foregut to hindgut in some phloem‐feeding hemipteran insects. In contrast, amino acids, proteins, and lipids are retained and digested in the midgut by digestive enzymes.^32^ The filtering function is generally postulated to involve combinations of passive osmosis/diffusion, hydrostatic pressure, and active transport by proton‐driven ATPases.^33^ Thus, the expression of *vATPase‐B* is required for the nutrient absorption in the midgut and osmoregulation in the filter chamber of *B. tabaci*.

Spraying the formulated dsRNA specific to *vATPase‐B* was effective to kill *B. tabaci* as well as *F. occidentalis*. The RNAi of *vATPase‐B* expression may impair the proton pumping activity and lead to malfunctioning of the enzyme, which interferes with nutrient absorption and osmoregulation of *B. tabaci* until death. We already demonstrated the insecticidal activity against *F. occidentalis* using the dsRNA specific to *vATPase‐B* of *F. occidentalis*.^19^, ^20^ This study showed a similar toxicity against the thrips using dsRNA specific to *vATPase‐B* of *B. tabaci*. The cross‐activity can be explained by the high sequence homology over 80% between two species and by the presence of the consecutively identical sequence fragments longer than 16 bp.^34^ Thus, the identical sequence fragments would produce the identical siRNA from RISC, which specifically bind to the target mRNA for RNAi in *F. occidentalis*. However, the dsRNA was not effective to control *B. tabaci* when it was sprayed without any formulation. This may be explained by poor delivery of dsRNA to the whiteflies because most sprayed dsRNAs might be on the surface of the hot pepper plants while the whiteflies are one of the sap feeders through phloem.^4^ To support this explanation, we expressed the dsRNA in the host plant using VIGS. VIGS technique uses a plant's antiviral defensive mechanism to suppress the expression of specific invasive viral transcripts.^35^ Our VIGS successfully expressed the cloned gene fragment from the control demonstration of the VIGS specific to PDS. By this RNAi, the target gene expression was significantly suppressed and phenotypically altered the leaf color. Under this condition, a partial fragment of *vATPase‐B* fragment was expressed using VIGS and confirmed by qPCR. When the whiteflies fed the VIGS‐treated hot peppers, they showed significant reduction in the target gene expression level and suffered from high mortality. Furthermore, the reduction of *vATPase‐B* transcript level was observed from FISH analysis in the midgut and filter chamber. These indicate that dsRNA specific to *vATPase‐B* of *B. tabaci* is toxic to both the whitefly and the thrips. Due to the high conserved nature of the *vATPase‐B* gene among species, it may be concerned of a potential of off‐target effects on non‐target organisms. Our previous study^21^ analyzed this off‐target effects in different dsRNAs produced from different locations and lengths designed from the *vATPase‐B* gene of *F. occidentalis*. The dsRNA used in this study was relatively less off‐target effects to predator and honeybee among the different dsRNAs generated from the *vATPase‐B* gene. A similar approach needs to be assessed in the predators and other non‐target organisms. In addition, RNAi efficacy can vary between laboratory and field populations due to sequence variation.^36^ In our current study, we used a field population of *B. tabaci* but a laboratory population of *F. occidentalis*. This suggest that the control efficacy of the dsRNA may be varied among field populations.

To be practical, the effective dsRNA delivery for *B. tabaci* used LDH formulation. This formulation of dsRNA was effective to control *B. tabaci* as well as *F. occidentalis*. Nanoparticle formulation of dsRNA such as chitosan or LDH has been useful to controlling the piercing‐sucking pests on herbaceous plants.^13^, ^17^ A recent study demonstrated a high efficiency of a chitosan nanoparticle of dsRNA specific to ecdysone receptor of *B. tabaci*.^37^ Indeed, our current study shows that these formulations significantly enhanced the control efficacy of *B. tabaci*, in which LDH formulation was superior to chitosan formulation. Cheng *et al*.^38^ compared four different nanoparticle formulations and demonstrated the superior property of LDH in its systemic delivery within the plant tissues and successful transfer to insect gut. Our current study also adds another merit of LDH in its long‐lasting efficacy because of its persistence on the leaf. This would allow us to decrease the application amount of dsRNA. Although the control efficacy increased with the treated dsRNA concentration at 7 days after treatment, the long‐lasting LDH efficacy increased the control efficacy in each control dose. The long‐lasting efficacy of the sprayed dsRNA in the hot peppers might be explained by facilitating dsRNA penetration and delivery in the plant tissues by LDH formulation. Cheng *et al*.^38^ showed that LDH formulation increased the adhesion of dsRNA onto leaf surface, facilitated its internalization into leaf cells, and delivered dsRNA from the stem to the leaf via the vascular system in the plants.

Altogether, our results show that spraying LDH‐formulated dsRNA specific to *vATPase‐B* of *B. tabaci* is effective to control both the whiteflies and the thrips infesting hot peppers cultivating in a glasshouse. To further enhance the control efficacy of sucking insect pests, alternative genes may be targeted by the dsRNA. For example, two genes of aquaporin and alpha‐glucosidase may be optimal targets to effectively control these sucking insects because these two genes are involved in maintaining osmotic homeostasis.^39^ In addition, this kind of multiple target dsRNA approach needs to consider the off‐target issue in the field conditions.

## CONFLICT OF INTEREST

The authors state that there are no conflicts of interest with any parties.

## Supporting information


**Table S1.** Primers used in this study.
**Table S2.** GenBank accession numbers of *vATPase‐B* genes used to construct a phylogeny tree in Fig. 1(C).
**Figure S1.** Molecular identification of *Bemisia tabaci* isolate. (A) Cytochrome oxidase I partial sequences of its DNA and protein. (B) Species identification (see arrow) among the species complex of *B. tabaci*.
**Figure S2.** Sequence alignment of *vATPase‐B* sequences of *Bemisia tabaci* and *Frankliniella occidentalis*.
**Figure S3.** Diagram of (A) chitosan and (B) LDH formulation.

## Data Availability

The data that support the findings of this study are available from the corresponding author upon reasonable request.
