# Supplementary material for: Control of two sucking insect pests, a whitefly ( Bemisia tabaci ) and a thrips ( Frankliniella occidentalis ), infesting hot peppers by spraying LDH‐formulated dsRNA
Source: Pest Manag Sci. 2025 Aug 11;81(12):8163–78. doi: 10.1002/ps.70125 (PMC12618903; doi:10.1002/ps.70125)
Supplement: Supplementary file 1 — Table S1. Primers used in this study. Table S2. GenBank accession numbers of vATPase‐B genes used to construct a phylogeny tree in Fig. 1(C). Figure S1. Molecular identification of Bemisia tabaci isolate. (A) Cytochrome oxidase I partial sequences of its DNA and protein. (B) Species identification (see arrow) among the species complex of B. tabaci. Figure S2. Sequence alignment of vATPase‐B sequences of Bemisia tabaci and Frankliniella occidentalis. Figure S3. Diagram of (A) chitosan and (B) LDH formulation. [file PS-81-8163-s001.docx]

**Supplementary Information**

**Table S1.** Primers used in this study

**Table S2**. GenBank accession numbers of *vATPase-B* genes used to construct a phylogeny tree in Fig. 1C

**Fig. S1.** Molecular identification of B. tabaci isolate. (A) Cytochrome oxidase I partial sequences of its DNA and protein. (B) **S**pecies identification (see arrow) among the species complex of *B. tabaci*

**Fig. S2.** Sequence alignment of *vATPase-B* sequences of *B. tabaci* and *F. occidentalis*

**Fig. S3.** Diagram of (A) chitosan and (B) LDH formulation

**Table S1.** Primers used in this study. ‘Fo’ and ‘Bt’ represent *F. occidentalis* and *B. tabaci*, respectively.

| Genes | Sequence (5’-3’) | Uses | Annealing temp (^0^C) | Expected size (bp) |
| --- | --- | --- | --- | --- |
| Bt-vATPase B | CATGAATTCCATTGCCCGTG | RT-PCR  RT-qPCR | 55.0 | 317 |
|  | ATTCAGCGGCTGTTAAGGCT |  |  |  |
| Fo-vATPase B | GATGAATTCTATTGCTCGTG | RT-PCR  RT-qPCR | 55.0 | 317 |
|  | ACTCAGCAGCAGTGAGAGCC |  |  |  |
| T7+ Bt-vATPase B | TAATACGACTCACTATAGGGAGACATGAATTCCATTGCCCGTG | RNAi | 55.0 | 363 |
|  | TAATACGACTCACTATAGGGAGAATTCAGCGGCTGTTAAGGC |  |  |  |
| Ca-PDS | AGGTCTTCTTTGGGAACTGAT | RT-PCR  RT-qPCR | 52.0 | 324 |
|  | GTTATGGAATGGGGATTACGA |  |  |  |
| Ca-β-actin | AATCAATCCCTCCACCTCTTCACTC | RT-PCR  RT-qPCR | 52.0 | 173 |
|  | CATCACCAGCAAATCCAGCCTT |  |  |  |
| Bt-vATPase B | Antisense-[FAM]ATTCAGCGGCTGTTAAGGCT | FISH | - | - |
|  | Sense-[FAM]AGCCTTAACAGCCGCTGAAT |  |  |  |
| Bt-Elongation Factor α  (Bt-EFα) | GCTGATTGTGCCGTGCTTATTG | RT-qPCR | 55.0 | 131 |
|  | ACGCGGTTGAGGGTGGTATT |  |  |  |
| Fo-Elongation Factor 1  (Fo-EF1) | TCAAGGAACTGCGTCGTGGAT | RT-qPCR | 55.0 | 160 |
|  | ACAGGGGTGTAGCCGTTAGAG |  |  |  |

**Table S2.** GenBank accession numbers of *vATPase-B* genes used to construct a phylogeny tree in Fig. 1C

| Organism name | GenBank accession number | Order |
| --- | --- | --- |
| *Bemisia tabaci* | XP_018896879.1 | Hemiptera |
| *Macrosteles quadrilineatus* | XP_054288745.1 |  |
| *Homalodisca vitripennis* | XP_046668751.1 |  |
| *Nilaparvata lugens* | XP_039298824.1 |  |
| *Planococcus citri* | XP_065223849.1 |  |
| *Frankliniella occidentalis* | KAE8747108.1 | Thysanoptera |
| *Frankliniella fusca* | KAK3914993.1 |  |
| *Thrips palmi* | XP_034236214.1 |  |
| *Megalurothrips usitatus* | KAJ1522819.1 |  |
| *Spodoptera exigua* | AQQ72786.1 | Lepidoptera |
| *Helicoverpa zea* | XP_047036044.1 |  |
| *Trichoplusia ni* | XP_026728890.1 |  |
| *Spodoptera frugiperda* | XP_035441832.1 |  |
| *Spodoptera litura* | XP_022827405.1 |  |
| *Ostrinia furnacalis* | XP_028172197.1 |  |
| *Neodiprion lecontei* | XP_015514003.1 | Hymenoptera |
| *Microplitis mediator* | XP_057339847.1 |  |
| *Microplitis demolitor* | XP_008548378.1 |  |
| *Sitophilus oryzae* | XP_030768315.1 | Coleoptera |
| *Dalotia coriaria* | XP_065173849.1 |  |
| *Cylas formicarius* | XP_060528829.1 |  |
| *Onthophagus taurus* | XP_022918076.1 |  |
| *Anabrus simplex* | XP_067007509.1 | Orthoptera |
| *Gryllus bimaculatus* | GLH04115.1 |  |
| *Schistocerca americana* | XP_046993029.1 |  |
| *Lucilia cuprina* | XP_023308965.1 | Diptera |
| *Aedes albopictus* | XP_019561805.1 |  |
| *Aedes aegypti* | XP_001651458.1 |  |
| *Bactrocera dorsalis* | XP_011212553.1 |  |
| *Calliphora vicina* | XP_065372203.1 |  |
| *Musca domesticas* | XP_005181053.1 |  |
| *Procambarus clarkii* | XP_069193256.1 | Crustacea |

**(A)**

>Seq1 [organism=Bemisia tabaci] Bemisia tabaci isolate ANU-TG mitochondrial cytochrome oxidase subunit 1 (CO1) gene, partial cds

TTGATTTTTTGGTCATCCAGAAGTTTATGTTCTTATTTTACCAGGGTTTGGAATTGTTTCTCATTTAATTAGCAGCGAGGCTGGAAAATTAGAGGTATTTGGAAGGTTGGGGATAATTTATGCTATATTGACTATTGGTATCTTAGGGTTTATTGTTTGAGGACATCATATATTTACAGTTGGAATAGATGTAGATACTCGAGCTTATTTCACTTCAGCTACTATGATTATTGCCGTTCCTACAGGAATTAAAATTTTTAGTTGGCTTGCTACTTTGGGTGGAATAAAGTCCAATAAATTCAGGCCCCTTGGCCTTTGATTTACAGGATTTTTATTTTTATTTACTATAGGTGGATTAACTGGAATTATTCTTGGTAACTCTTCTGTAGATGTGTGTTTGCATGACACTTATTTTGTTGTTGCGCATTTTCATTATGTCTTATCAATAGGAATTATTTTTGCTATTGTAGGAGGAGTTATCTATTGATTTCCATTAATCTTGGGCTTAACCTTAAATAATTATAGCTTGGTGTCTCAATTTTATATCATGTTCATTGGAGTAAATTTAACTTTTTTTCCTCAGCATTTTCTTGGTTTGGGGGGAATGCCTCGCCGATATTCAGATTATGCTGATTGTTATCTAGTATGGAACAAAATTTCTTCTGCGGGAAGGATTTTGAGTATCATTTCTGTTATTTATTTTTTATTTATTGTTTTAGAATCTTTTCTTCTTTTGCGTTTAGTAAGATTTAAGCTTGGTGTAAGCAGACATCTAGAATGGAAAATTAATAAACCAGCCCTTAATCACAGTTTTAAAGAGTTGTGTTTAATTTTTTTTTTC

>Seq1 [organism=Bemisia tabaci] Bemisia tabaci isolate ANU-TG mitochondrial cytochrome oxidase subunit 1 (CO1) gene, partial cds

WFFGHPEVYVLILPGFGIVSHLISSEAGKLEVFGSLGMIYAMLTIGILGFIVWGHHMFTVGMDVDTRAYFTSATMIIAVPTGIKIFSWLATLGGMKSNKFSPLGLWFTGFLFLFTMGGLTGIILGNSSVDVCLHDTYFVVAHFHYVLSMGIIFAIVGGVIYWFPLILGLTLNNYSLVSQFYIMFIGVNLTFFPQHFLGLGGMPRRYSDYADCYLVWNKISSAGSILSIISVIYFLFIVLESFLLLRLVSFKLGVSSHLEWKINKPALNHSFKELCLIFFF

**(B)**
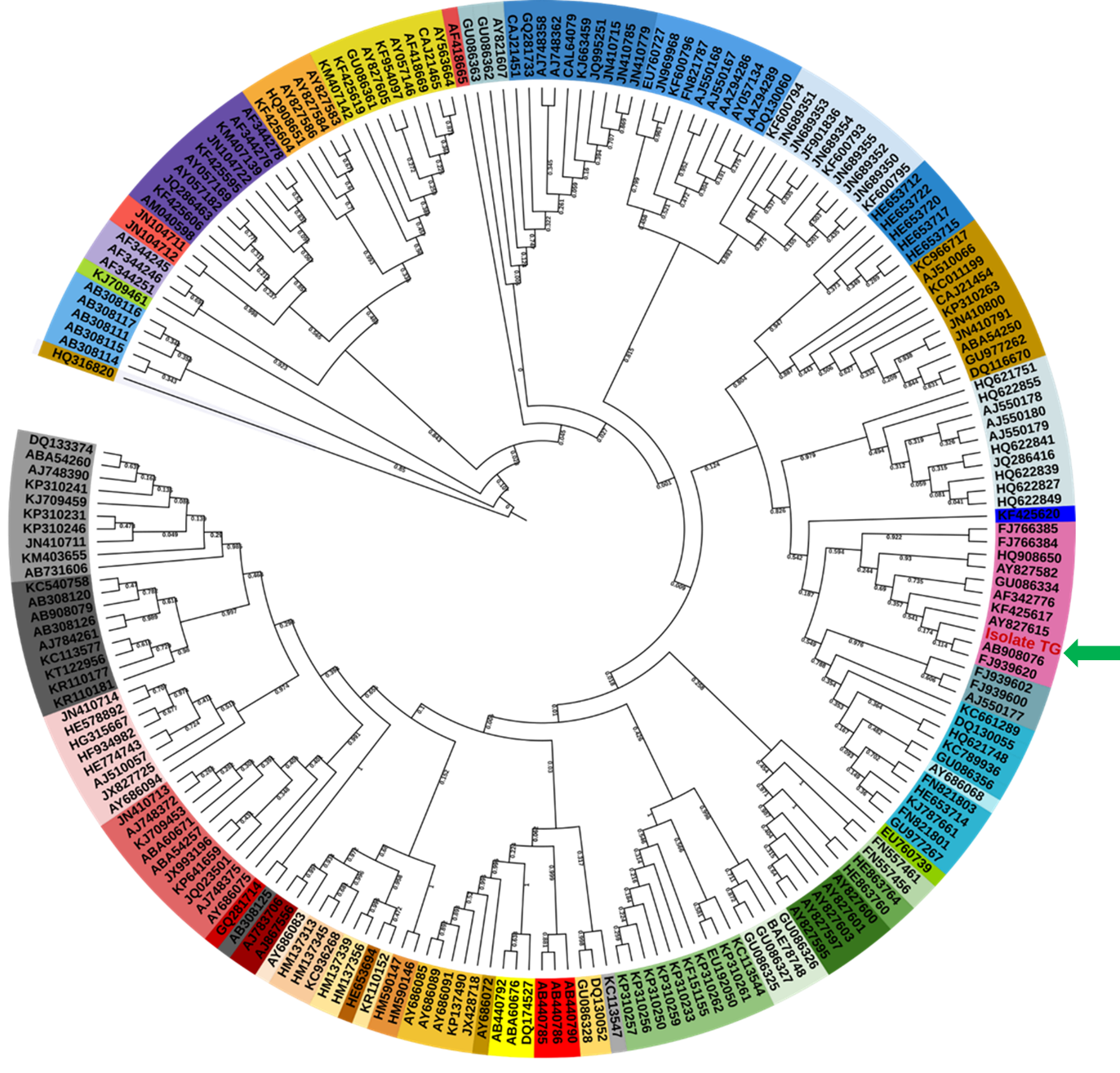


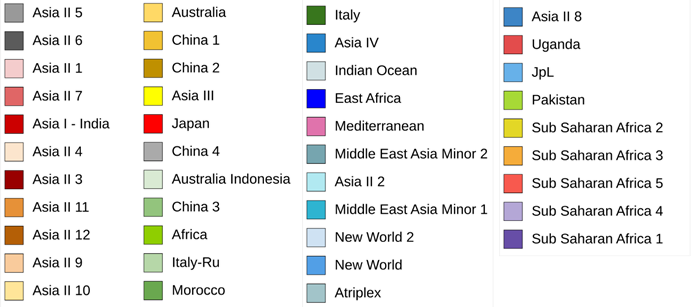


**Fig. S1**

**
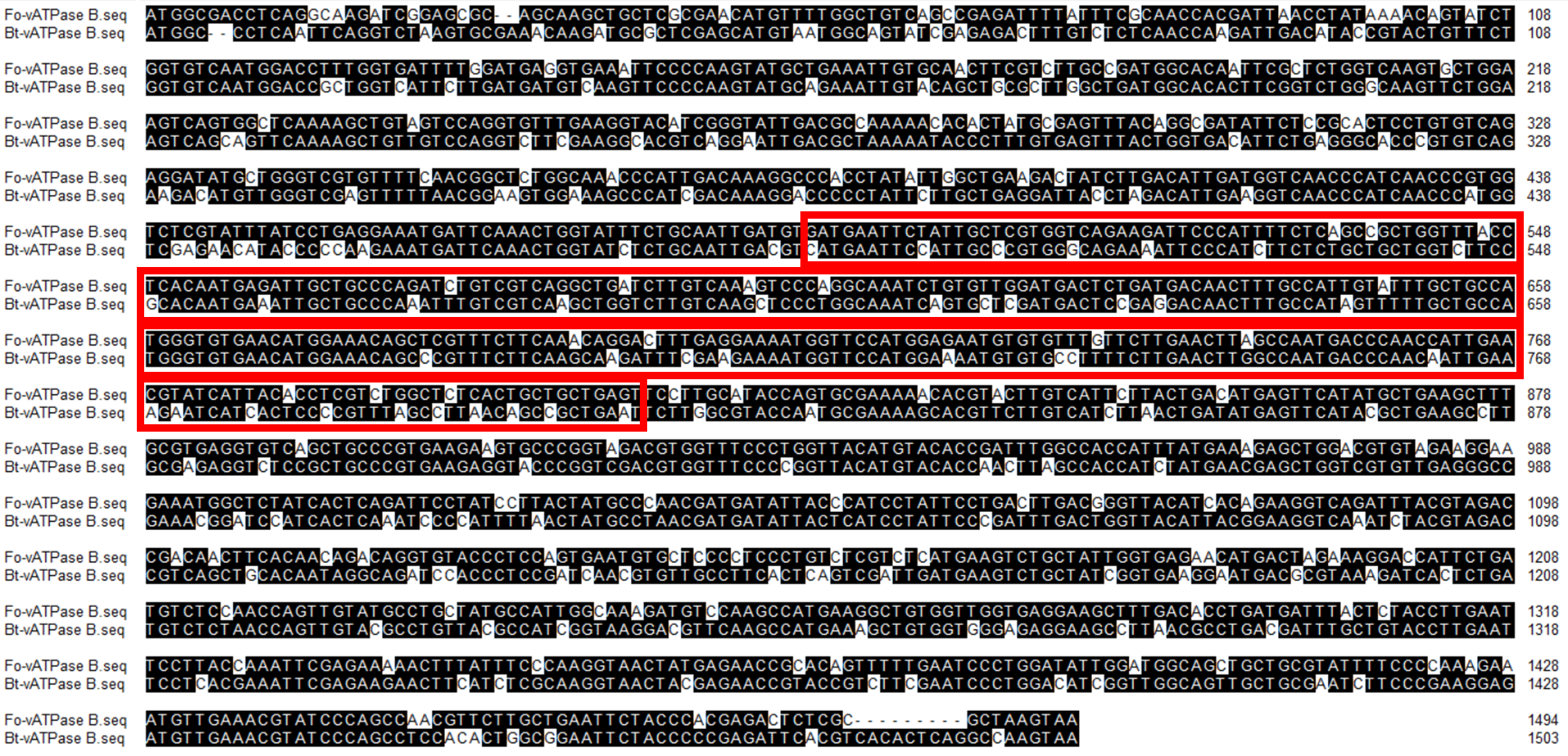
**

**Fig. S2**

**(A)**


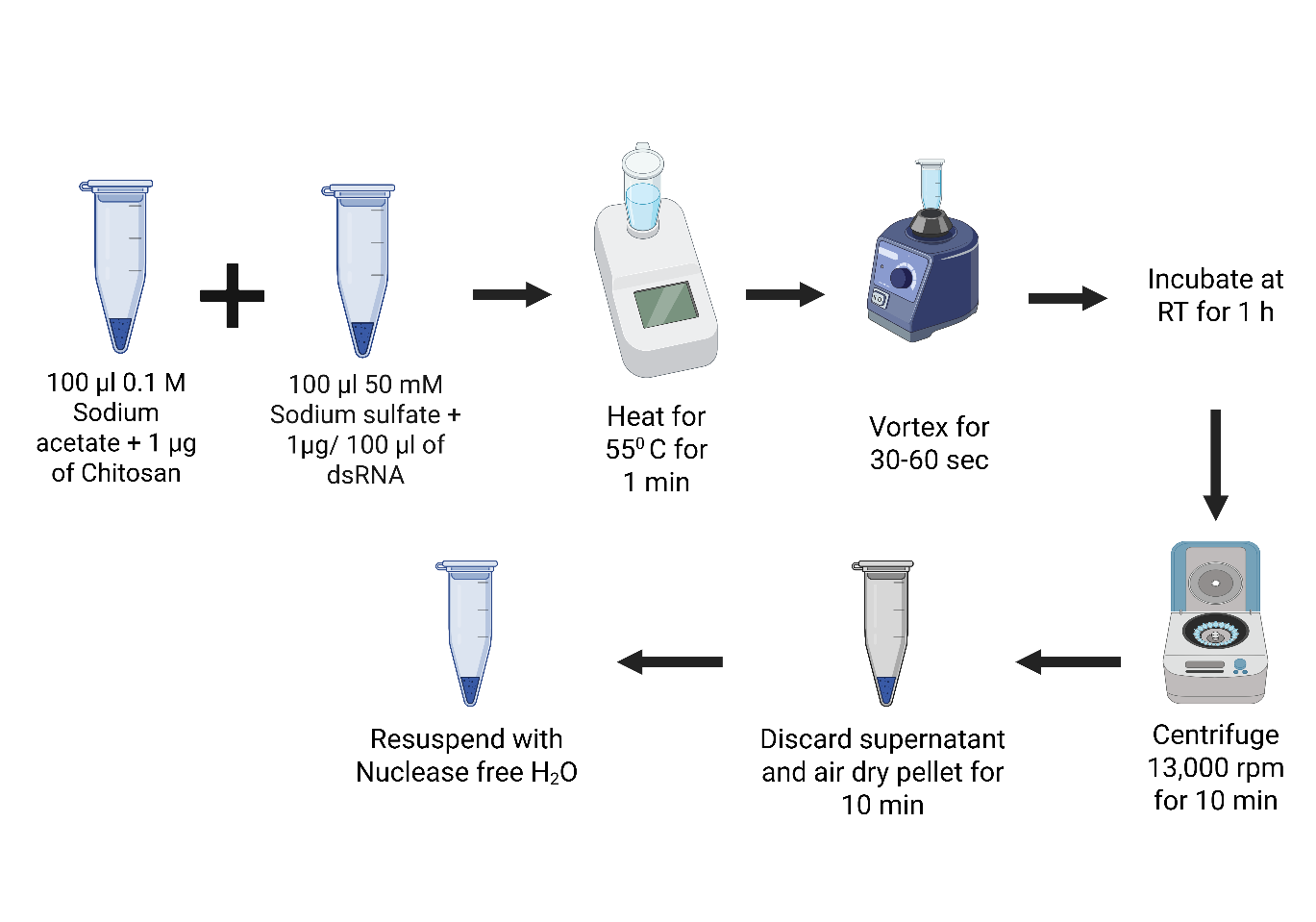


**(B)**


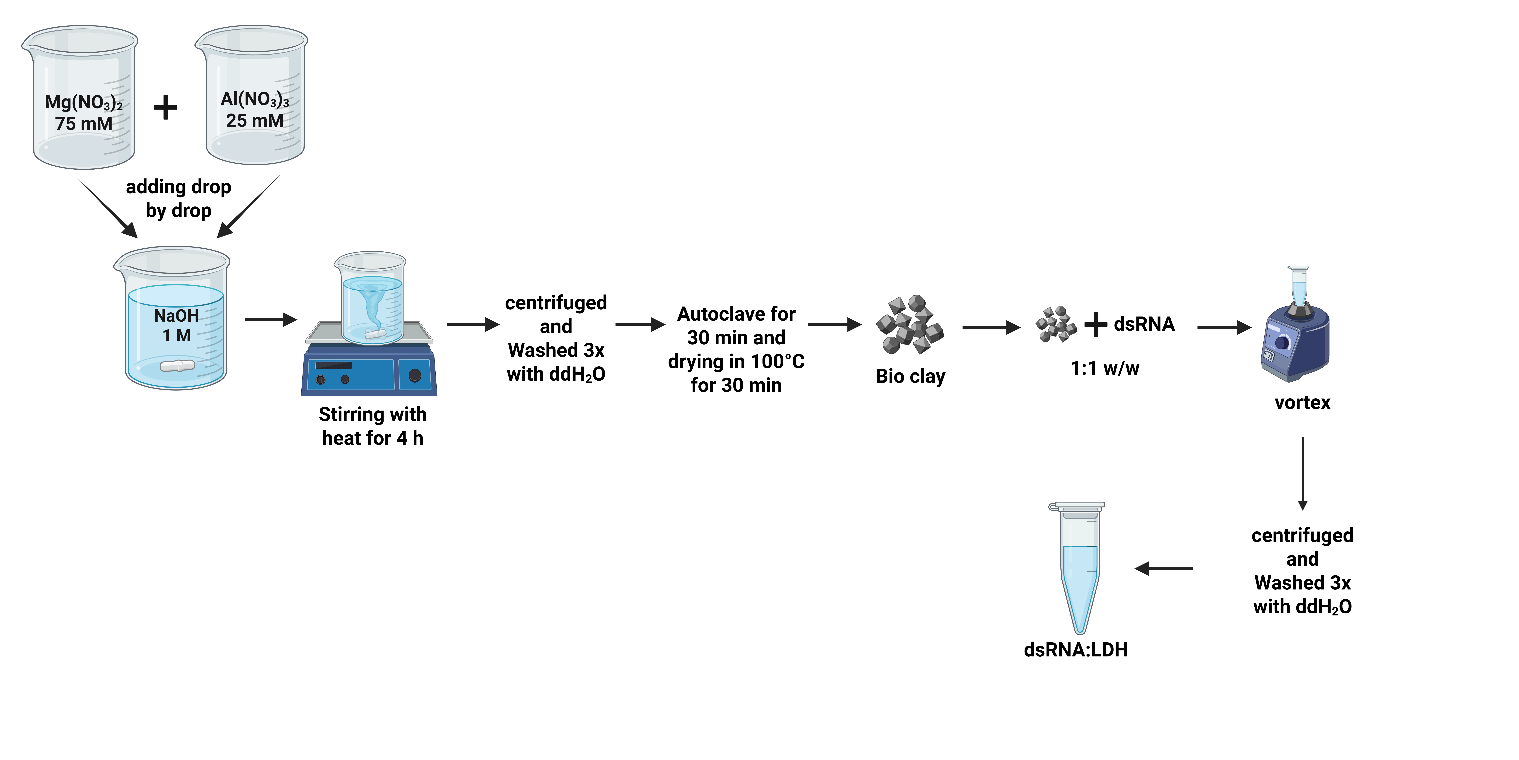


**Fig. S3**
